# Investigation on the α/δ Crystal Transition of Poly(l-lactic Acid) with Different Molecular Weights

**DOI:** 10.3390/polym13193280

**Published:** 2021-09-26

**Authors:** Lei Zhang, Guoqun Zhao, Guilong Wang

**Affiliations:** Key Laboratory for Liquid-Solid Structural Evolution and Processing of Materials (Ministry of Education), Shandong University, Jinan 250061, China; zhanglei_mail@sdu.edu.cn (L.Z.); guilong@sdu.edu.cn (G.W.)

**Keywords:** Poly(l-lactic acid), crystallization, α form

## Abstract

Poly(l-lactic acid) (PLLA) crystal possesses a complex polymorphism, and the formation mechanism of various crystal forms has been a hot research topic in the field of polymer condensate matter. In this research, five kinds of PLLA with different molecular weights were prepared by ring-opening polymerization with strict dehydration operations and multistep purification treatments. Then, thin film isothermal crystallization experiments were carried out to obtain crystallized samples. Previous research has proven that the PLLA α crystal form is usually formed at a temperature above 120 °C and the PLLA δ (or α’) crystal form is usually formed at a temperature below 120 °C. However, in this research, the characterization results indicated that the PLLA crystal changed from δ form to α form with the decrease of molecular weight at a temperature of 80 °C. Considering the molecular weight effect, the paper argued that the transitions of the α/δ crystal form are not only associated with temperature, but also related to entanglement state before crystallization. The small-angle X-ray scattering of the PLLA crystal and rheology analysis of the PLLA melt before crystallization further proved the significant role of entanglement. Finally, we tentatively proposed the entanglement effect mechanism on the transitions of the α/δ crystal form.

## 1. Introduction

Poly(l-lactic acid) (PLLA) is one of the most popular degradable and biocompatible polymers which can be produced from renewable resources, such as corn starch and sugar cane [1]. As a typical semicrystalline polymer, the mechanical properties [2] and service performances [3] of PLLA products strongly depend on the crystal structure of the PLLA.

PLLA possesses a complex polymorphism in its crystalline region. It is well known that PLLA crystal modifications mainly include the α form [4,5,6], δ (or α′) form [6,7,8], β form [6,9,10,11,12], and γ form [13]. Among these crystal forms, α and δ are the two most common forms of PLLA crystal. The unit cell of the α-form crystal belongs to an orthogonal system, with a = 10.68 Å, b = 6.17 Å, and c = 28.86 Å [6]. The helical chains of (10/7) conformation (10/7 for PLLA, 10/3 for PDLA [13,14]) are packed regularly at the corner and center positions of the unit cell [5,8,14,15]. The δ form also has the orthogonal unit cell of a = 10.80 Å, b = 6.20 Å, and c = 28.80, which is generated by annealing the melt-quenched sample at a relatively low temperature, below 120 °C [6,7,8]. The chain-packing mode of the δ form is similar to that of the regular α form, but the two (10/7) helical chains in δ unit cells are conformationally disordered [6].

The transition between α and δ crystal forms plays an important role in condensed matter evolution of PLLA. Zhang et al. [16,17] reported that the X-ray fiber pattern and polarized IR/Raman spectra were taken successfully for the uniaxially oriented δ form of PLLA, and they suggested that there are slight differences in both the chain-conformation and chain-packing mode between the α and δ forms. Zhang et al. [18] further investigated δ-to-α phase transition by simultaneous measurements of WAXD and DSC, and they found that the δ phase transforms discretely to the α phase in the first-order transition mode with chain packing of the crystal lattice becoming more compacted. Moreover, Pan et al. [19,20,21] also systematically studied the influence mechanism of molecular weight and annealing temperature on the δ-to-α transition mechanism of PLLA crystal. The interesting thing is that they found the molecular weight significantly affects the crystallization kinetics, while the polymorphism of PLLA is not significantly influenced by molecular weight. This conclusion was obtained from the crystallization experiments of PLLA with molecular weights of 15,000~218,000 g/mol [19,20]. So, for PLLA with a smaller molecular weight (<15,000 g/mol), does molecular weight still not affect the crystal form?

To study this problem, narrowly dispersed and highly pure PLLA with a small molecular weight should be prepared. In this work, we prepared PLLA with five different molecular weights by using ring-opening polymerization with strict dehydration operation and multistep purification treatments. Subsequently, crystals were obtained by film crystallization experiments of the PLLA samples with different crystallization temperatures. The crystal structures of PLLA δ crystal and α crystal were studied by using wide angle X-ray diffraction, Fourier transform infrared spectroscopy, differential scanning calorimetry, and small-angle X-ray scattering. The effect of molecular weight on the entanglement state of molecular chains in PLLA melt before crystallization was analyzed by a rheological test. Combined with the crystal structure and melt state of PLLA, the transition mechanism of δ/α was proposed.

## 2. Materials and Methods

### 2.1. Materials

The pristine PLLA samples (named as PLLA1, PLLA2, PLLA3, PLLA4, and PLLA5) were synthesized by ring-opening polymerization, using n-dodecanol and stannous caprylate as initiators and catalysts, respectively. Please see Section S2 of the Supplementary Materials for a detailed synthesis method. Before crystallization, the PLLA samples were purified by reprecipitation to eliminate residual l-lactide monomers or lactic acid monomers which may have been embedded in the crystal or amorphous phase. The detailed purification method is given in the Supplementary Materials and our previous research [22,23,24].

The number-average molecular weight $M_{n}$ and polydispersity $M_{w}/M_{n}$ of the purified samples were determined by gel permeation chromatography. The specific rotation value [α] was measured by using a spectropolarimeter. The glass transition temperature $T_{g}$ and melting point $T_{m}$ were measured by using a differential scanning calorimeter. The molecular weight, thermal properties, and specific rotation degree of five PLLA samples are listed in Table 1, and the original data of the material properties is given in Figures S1–S3 of the Supplementary Materials.

### 2.2. Methods

Confocal Laser Scanning Microscopy (CLSM). The altitude and morphology of the polymer film were measured by CLSM on a Zeiss LSM 700 (Carl Zeiss, Jena, Germany) at atmospheric condition, which can take the place of AFM when the size of the observed object is bigger than 50 μm.

Atomic Force Microscopy (AFM). AFM (Dimension Icon, Veeco, NY, USA) was used to measure the thickness and morphology of the crystals at atmospheric conditions, operating with silicon cantilevers in the PeakForce Tapping mode. The peak force error image was obtained to reflect the phase morphology of polymer samples.

Wide-Angle X-ray Diffraction (WAXD). WAXD measurement was performed by using a DMAX-2500PC (Rigaku Corporation, Tokyo, Japan) with a Cu Kα source, operating at 40 kV and 150 mA. The scanning range was from 5° to 35° and the rotate rate was 2°/min.

Differential Scanning Calorimeter (DSC). The melting processes of the crystallized PLLA samples were measured by using a NETZSCH STA 4495F5 apparatus (NETZSCH, Selb, Germany). The crystallized samples with an average weight of 5 mg were reheated to 200 °C at a rate of 10 °C/min under a nitrogen atmosphere.

Small-Angle X-ray Scattering (SAXS). SAXS (SAXSess mc^2^, AntonPaar, Graz, Austria) was used to measure the long periods, thickness of crystal layers, and thickness of amorphous layers of the lamellar stacks of PLLA samples with different molecular weights. The PLLA samples were stripped from the Si wafer after crystallization treatments. The operation voltage was 40 kV, and the operation current was 50 mA. The wavelength of X-ray radiation was 0.1542 nm, and the distance from sample to detector was 264.5 mm. The raw SAXS data were then background corrected according to a standard procedure.

Rotational Rheometer. A rotational rheometer (HAAKE MARS 40, Thermo Fisher Scientific, Waltham, MA, USA) was used to characterize the rheological properties of the PLLA samples. The testing samples for rheology characterization had a circle shape with a diameter of 20 mm and a thickness of 1.2 mm. The frequency sweep tests in a constant strain mode were performed at a strain of 0.1% over a frequency range of 0.1–10 Hz.

## 3. Results

### 3.1. Crystal Morphology of PLLA with Different Molecular Weights

**Figure 1:**
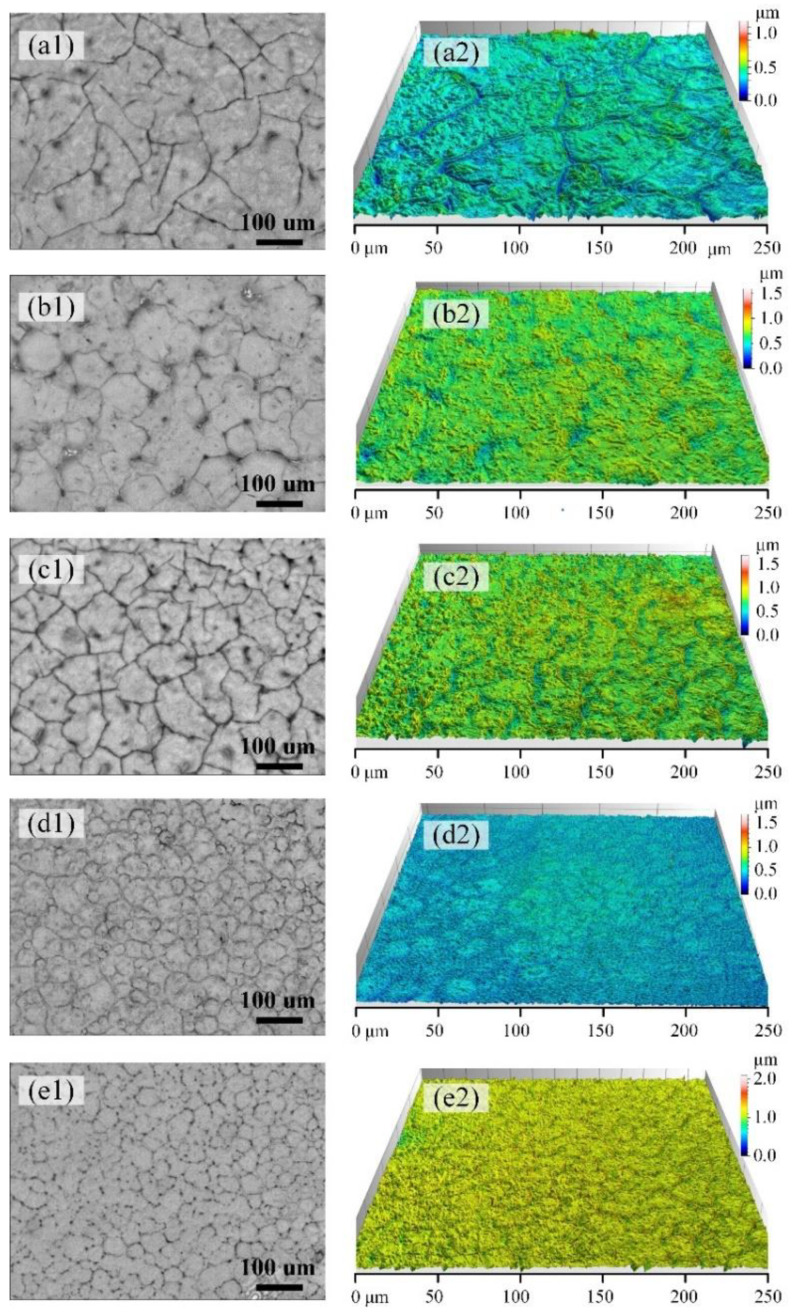
Morphology images of PLLA samples after crystallization treatment at a temperature of 80 °C: (**a1**–**e1**) show the microscopy images of PLLA1, PLLA2, PLLA3, PLLA4 and PLLA5, respectively. The three-dimensional images of (**a1**–**e1**) were obtained by CLSM and are shown in (**a2**–**e2**), respectively.

It is well known that molecular weight has significant influence on polymer crystallization. Five kinds of PLLA with different molecular weights were made into thin films, and then isothermal crystallization experiments were carried out. Please refer to Sections S3 and S4 of the Supplementary Materials for the methods of film preparation and crystallization treatment. The film thickness is measured as about 180 nm by AFM as shown as Figure S4. By using the in situ microscopy system and CLSM, the crystal morphologies formed at 80 °C are recorded and given in Figure 1. In Figure 1, with the increase of molecular weight, the size of the PLLA crystals decreases, and the nucleation density of the PLLA crystals (crystal number per unit area) increases, correspondingly. Due to the high mobility of the molecular chain, PLLA with a lower molecular weight starts to crystallize at a higher temperature. At this time, the supercooling degree of the system is low, and there are few crystallization centers induced by the heat fluctuation of the PLLA. For PLLA with a higher molecular weight, the mobility of its molecular chain segment is greatly weakened, the temperature range of the crystallization process is shifted to a low temperature, the system has a higher supercooling degree during crystallization, and the number of crystallization centers induced by the heat fluctuation of the PLLA is large.

**Figure 2:**
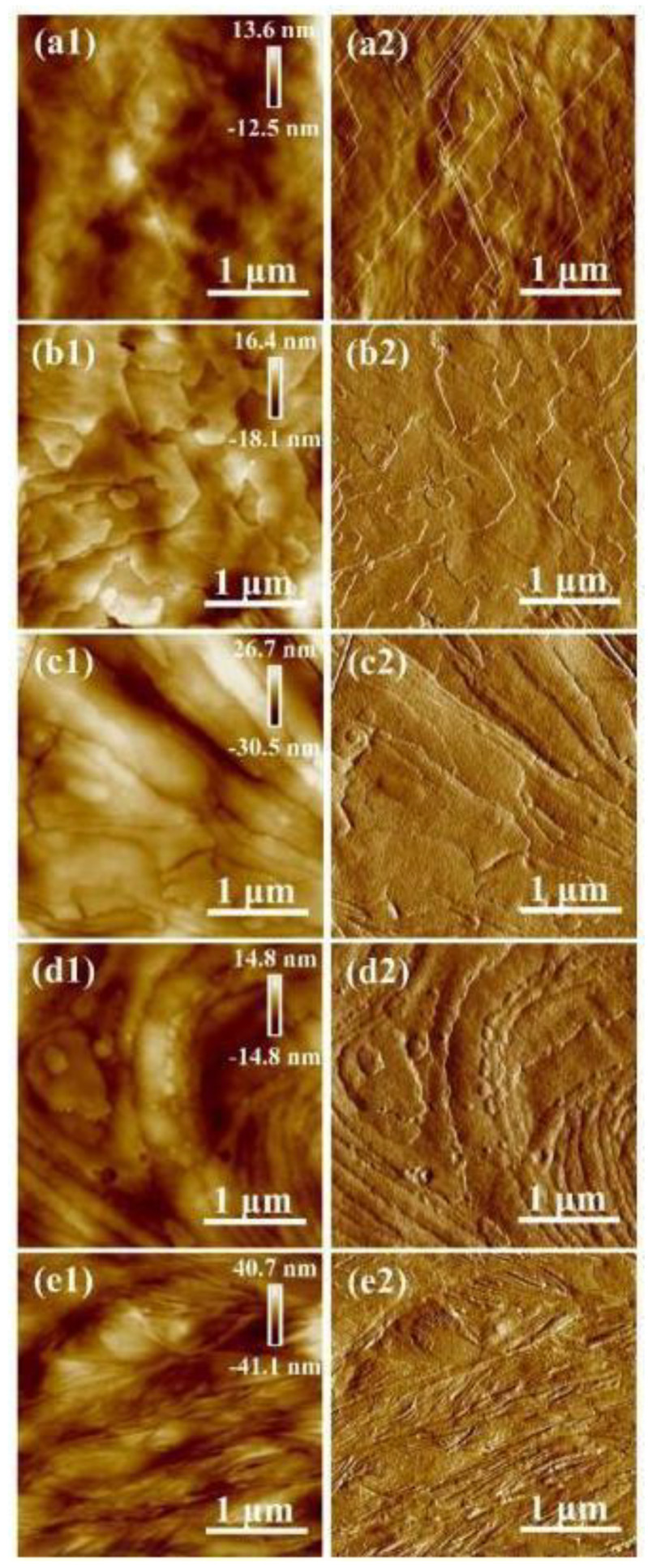
AFM images of the crystal surface of PLLA samples after crystallization treatment at a temperature of 80 °C: (**a1**–**e1**) show the height images of PLLA1, PLLA2, PLLA3, PLLA4 and PLLA5, respectively; (**a2**–**e2**) show the peak force error images of the five PLLA samples, respectively.

Figure 2 shows the AFM morphology of the crystal surface of the PLLA samples after crystallization treatment at a temperature of 80 °C. From the height images and peak force error images of the crystal surfaces, the PLLA1 and PLLA2 samples present many lamellar crystals with a relatively straight crystal boundary. The lamellar crystals are also found in the PLLA3 and PLLA4 samples. Compared to the boundary of the lamellar crystal in PLLA1 and PLLA2, the boundary of the lamellar crystal in PLLA3 and PLLA4 is irregular or curved. In addition, the PLLA5 crystals present a radial fibrous shape. According to the research of Forrest et al. [25], polymers with a low molecular weight prefer to form an observable crystallographic structure. In our previous work [23], the experiment results show that the amorphous chain segments outside the lamellar crystal can curve the crystal in PLLA with a high molecular weight. Based on Figure 1 and Figure 2, the molecular weight plays a dominant role in crystal morphology.

### 3.2. Form Transformation of PLLA Crystals

**Figure 3:**
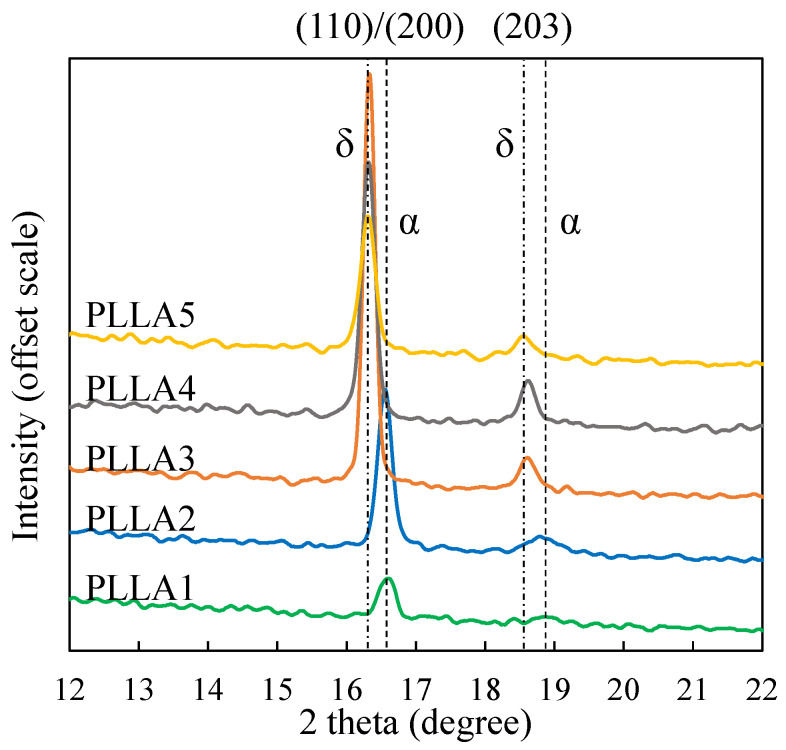
WAXD diffraction profiles of five PLLAs after crystallization treatment at a temperature of 80 °C.

This section will further investigate the crystal structure differences of the five PLLA samples. Figure 3 gives the WAXD diffraction profiles of the five PLLA samples after crystallization treatment at a temperature of 80 °C. In Figure 3, there are two kinds of curves. The curves of the PLLA3, PLLA4 and PLLA5 samples basically match that of the PLLA δ form [6,7,8], and the peaks are mainly found at 2θ ≈ 16.3° and 18.6°. According to Zhang et al. [7,16] and Pan et al. [19,20], the disordered α form (δ form) of PLLA is commonly acquired at a crystallization temperature below 120 °C. The crystallization temperature of PLLA5 was 80 °C, which conforms to the formation condition of the δ form. The curves of the PLLA1 and PLLA2 samples basically match that of the PLLA α form, whose dominant peak (110/200 reflection) is located at 2θ ≈ 16.7°, and the secondary peak (203 reflection) is located at 2θ ≈ 18.9°. Here, α crystals are formed at 80 °C from PLLA samples with molecular weights of 1700 and 5500 g/mol. This result means that the crystallization temperature condition (<120 °C) is not the only necessary condition to form PLLA δ crystals, and that changing molecular weight can also induce α/δ crystal form transition.

**Figure 4:**
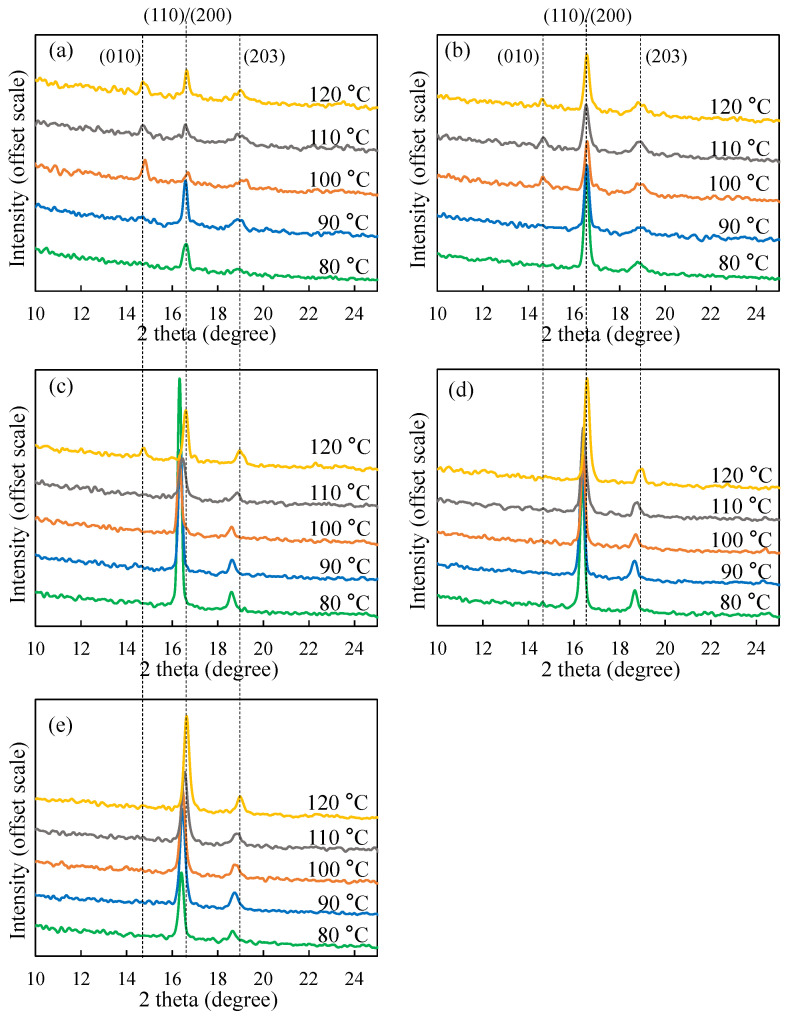
WAXD curves of five PLLA samples obtained by crystallization at different temperatures: (**a**) PLLA1; (**b**) PLLA2; (**c**) PLLA3; (**d**) PLLA4; (**e**) PLLA5.

Figure 4 shows the WAXD curves of PLLA samples treated at five temperatures of 80 °C, 90 °C, 100 °C, 110 °C and 120 °C. It can be seen from Figure 4 that the influence of temperature on the crystal form of PLLA is also obvious. From Figure 4a,b, with the increase of temperature, the location of dominant peaks representing the 110/200 reflection remain unchanged, and the peaks representing the 010 reflection become more and more obvious. As shown in Figure 4c–e, with the increase of temperature, the 2θ of dominant peaks representing the 110/200 reflection of PLLA3-PLLA5 samples shifts to a higher value. The higher 2θ value of the WAXD peaks at this location indicates the α/δ crystal form transition of the PLLA crystal. In this way, increasing crystallization temperature can cause the crystal form to change from δ to α when the molecular weight is larger than that of PLLA3 (≈10,800 g/mol). For the PLLA1 and PLLA2 sample, only α crystals are found in the temperature range of 80–120 °C. Furthermore, the FTIR results given by Figure S5 in the Supplementary Materials also support the judgment about α and δ crystals based on the previous studies [26,27,28,29]. Next, we will further study the calorimetric properties of these two kinds of crystal forms by DSC.

In Figure 3, the main peak of WAXD curve obtained by samples PLLA1 and PLLA2 after crystallization at 80 °C conforms to the common peak positions of α crystals, but there is no diffraction peak corresponding to the (010) crystal plane at 14.4°. In addition, WAXD curves of α crystals of the PLLA4 and PLLA5 samples at 14.4° also did not show the diffraction peak corresponding to the (010) crystal plane. Hence, combining Figure 3 and Figure 4, it can be concluded that when the molecular weight of the PLLA is low, or the crystallization temperature is high, the WAXD curve will have a diffraction peak at 14.4°. Similar results have been obtained by Zhang and Tashiro et al. [17]. Zhang found that when the crystallization temperature is above 140 °C, the diffraction peak corresponding to the (010) crystal plane will appear at 14.4°, and with the increase of crystallization temperature, the diffraction peak at 14.4° becomes more and more obvious. In addition, the WAXD data of Pan and Inoue et al. [20] showed that the diffraction peak at 14.4° became more and more obvious with the increase of annealing time at 150 °C. In this way, it can be inferred that the diffraction peak will appear at 14.4° when the PLLA α crystal is highly ordered. In this study, the PLLA samples in Figure 3 were crystallized at 80 °C. Although α crystal was obtained, the WAXD curve did not show a diffraction peak at 14.4°, due to the low crystallization temperature.

### 3.3. Calorimetric Properties of α and δ Crystal

**Figure 5:**
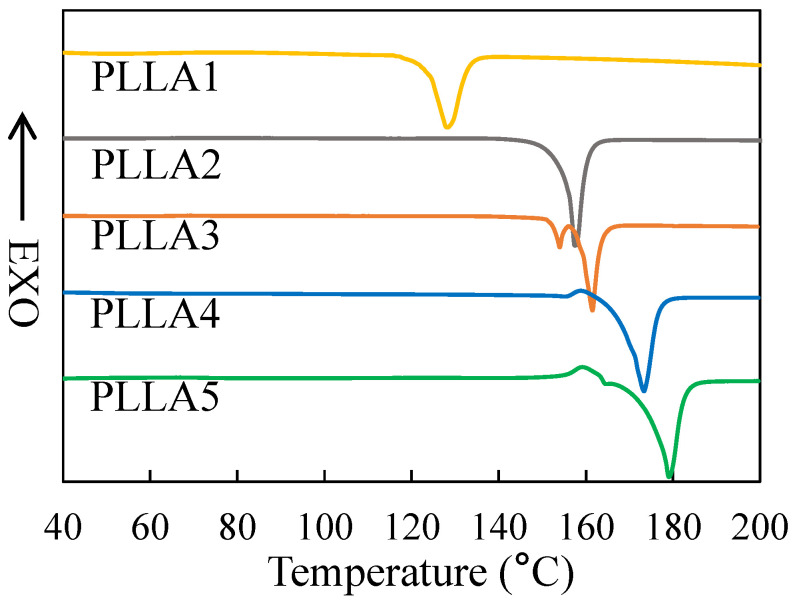
DSC melting curves of PLLA samples after isothermal crystallization at 80 °C. The exothermal direction is marked on the *Y*-axis.

Figure 5 gives the DSC melting curves of the PLLA samples after crystallization at 80 °C. Combining with the crystal form given by WAXD, the melting peaks of PLLA1 at 128.3 °C and of PLLA2 at 158.6 °C indicate the melting processes of α crystals, and the melting peaks of PLLA4 at 173.4 °C and of PLLA5 at 178.9 °C mainly indicate the melting processes of δ crystals. Moreover, the peak shapes of PLLA1 and PLLA2 show that the α crystal is directly melted without other form transition or recrystallization, while in the DSC curves of PLLA4 and PLLA5, a small exothermal peak around 160 °C appears prior to the melting point. According to Zhang’s research [7], the small exothermal peak corresponds to the disorder-to-order (δ-to-α) phase transition, in which the chain packing of the crystal lattice becomes more compact. This phenomenon demonstrates that the δ crystal is the main crystal form in the PLLA4 and PLLA5 samples.

The DSC curve of PLLA3 shows two melting peaks at 154.0 °C and 161.6 °C, respectively. This kind of double melting peak of PLLA was also found in other research [18,19]. According to this research, the left endotherm peak includes two contributions: (i) the melting of the initial δ crystal and (ii) the recrystallization into the α crystal from the melted δ crystal, which results in the endotherm and exotherm in the DSC curves, respectively. So, the peak at 154.0 °C may be an overlapping peak of the endotherm and exotherm.

Furthermore, there is significant difference among the peak positions of the same crystal form, e.g., the melting peaks of PLLA1 and PLLA2. According to the Gibbs–Thomson equation for melting point, the deviation of melting point temperature Tm from the thermodynamic equilibrium melting point $T_{m}^{0}$ can be derived by [30]:

(1) $$T_{m}-T_{m}^{0}=1-\frac{2\sigma_{e}}{d_{c}\Delta h}$$

where $\sigma_{e}$ represents the end surface free energy, Δh is the melting enthalpy and $d_{c}$ is the lamellar thickness. Because $\sigma_{e}$ and Δh of a certain crystal form are almost constant, the melting points obtained from DSC heating processes are mainly influenced by lamellar thickness. To investigate crystal thickness, SAXS measurement was further carried out.

### 3.4. SAXS Analysis of PLLA Samples

**Figure 6:**
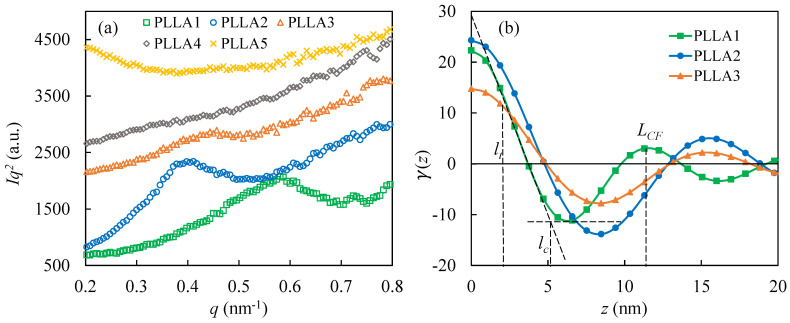
SAXS measurement results of crystallized PLLA samples after crystallization treatment at 80 °C: (**a**) Lorentz-corrected SAXS profiles; (**b**) one-dimensional correlation function profiles of PLLA1, PLLA2 and PLLA3 samples. The correlation function long periods ($L_{CF}$), crystal thicknesses ($l_{c}$) and transition region thicknesses ($l_{t}$) are marked on PLLA1′s curve as an example.

Figure 6 shows the SAXS measurement results of crystallized PLLA samples with different molecular weights. The Lorentz-corrected one-dimensional scattering intensity distributions of the five PLLA samples are shown in Figure 6a. In Figure 6a, the horizontal axis is labeled by a scattering vector q (q=4πsinθ/λ, where θ is half of the scattering angle, λ is the wavelength of the X-ray), and the vertical axis is labeled by $Iq^{2}$, the scattering intensity. The essence of small-angle scattering is caused by the difference of electron cloud density in the sample. In Figure 6a, the PLLA1 and PLLA2 samples have obvious scattering peaks $q_{max}$ at 0.58 nm^−1^ (Bragg long periods: $L_{B}=2\pi /q_{max}$ = 10.83 nm) and 0.39 nm^−1^ (Bragg long periods: $L_{B}$ = 16.11 nm), respectively. The PLLA3 sample has a relatively weak scattering peak at 0.43 nm^−1^, and the Bragg long periods can be calculated as 14.61 nm. Different from the PLLA1 and PLLA2 samples, it is hard to find the scattering peak of the Lorentz-corrected SAXS profiles of the PLLA4 and PLLA5 samples. This may be caused by the disordered crystal structure of the δ crystal, which does not have enough difference in electron cloud density between the crystalline and amorphous regions. These SAXS results were also supported by Pan et al., who found that no discernible peak is observed on the Lorentz-corrected SAXS profiles of PLLA crystallized at a temperature lower than 90 °C [21].

In the lamellar stack model with sharp phase boundary, the long period represents the sum of the crystal thickness ($l_{c}$) and the amorphous layer thickness ($l_{a}$). The one-dimensional correlation function is utilized here to further calculate the $l_{c}$ and $l_{a}$ [31,32]. The one-dimensional correlation function is given by [32]:

(2) $$\gamma \left(z\right)=\frac{\int_{0}^{\infty }I\left(q\right)q^{2}cos\left(qz\right)dq}{\int_{0}^{\infty }I\left(q\right)q^{2}dq}$$

where z is the direction normal to the lamellar interface. Figure 6b shows the calculation results of the one-dimensional correlation function of the PLLA1, PLLA2 and PLLA3 samples. Because PLLA4 and PLLA5 have no obvious scattering peak within 0.2–0.8 nm^−1^ of the scattering vector q, the corresponding one-dimensional correlation function cannot be given. The correlation function long period $L_{CF}$ can be obtained by locating the first maximum in the one-dimensional correlation function [31,32]. The crystal thickness ($l_{c}$) is given by the intersection between the straight line extended from the self-correlation triangle and the baseline in the one-dimensional correlation function [33], as shown as Figure 6b. The amorphous layer thickness ($l_{a}$) is then given by $l_{a}=L_{CF}-l_{c}$. Moreover, there is a transition region between the crystalline region and the amorphous region. Based on Strobl’s theory [34], the transition region thickness ($l_{t}$) can be given by the horizontal length of the curved portion of the hypotenuse of the autocorrelation triangle, as shown as Figure 6b. According to the above calculation methods, Table 2 lists the SAXS data analysis results of the PLLA1, PLLA2, and PLLA3 samples.

From the data listed in Table 2, for the crystals in the PLLA1, PLLA2, and PLLA3 samples, the long period and crystal region thickness of the different samples show a similar size change trend. The thickness of α crystals in PLLA2 is obviously larger than that of PLLA1. This difference of crystal thickness can explain the difference in melting temperature of the PLLA1 and PLLA2 samples shown in Figure 5.

It should be noted that some δ crystal form of PLLA still can be detected by SAXS. For example, the δ crystal formed at a temperature of 100 °C reported by previous research [21]. In this research, a weak peak is also observed on the Lorentz-corrected profile of the PLLA3 δ crystal, which is characterized by WAXD (Figure 3). Why can SAXS perceive the δ crystal in the PLLA3 sample, while not the δ crystal in the PLLA4 and PLLA5 samples? The following discussion may give an answer.

### 3.5. Rheology Properties of PLLA

**Figure 7:**
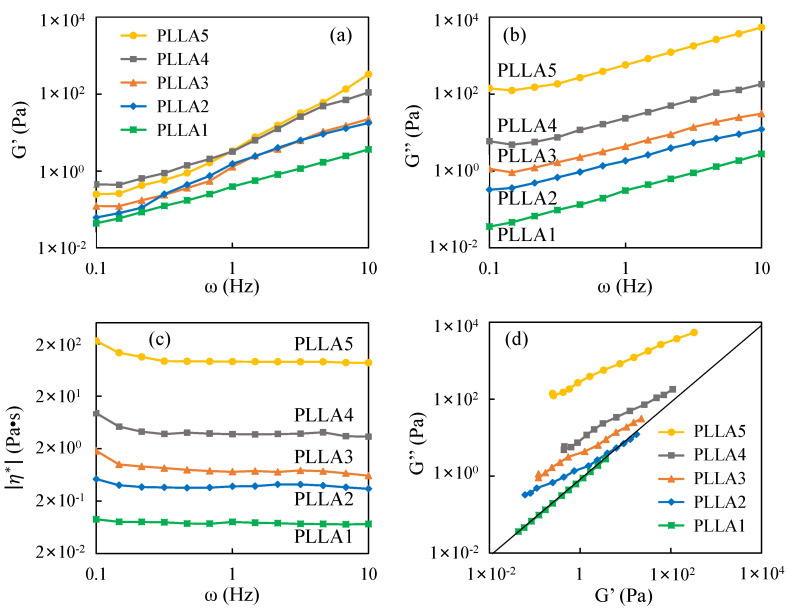
The dynamic frequency scanning curves of five PLLA samples at 190 °C: (**a**) storage modulus G′ vs. frequency ω; (**b**) loss modulus G″ vs. frequency ω; (**c**) complex viscosity $\left|\eta^{*}\right|$ vs. frequency ω; (**d**) storage modulus G′ vs. loss modulus G″.

To understand the mechanism of α/δ crystal form transition in PLLA crystals, the melt state before crystallization should be considered. In the polymer crystallization process, the melt state can be retained to the growth front of the crystal, thereby affecting crystallization. Wang et al. characterized the entanglement state of iPP by rheological testing, and then studied the crystallization behavior of iPP-disentangled melt [35]. R. Kurz et al. also related the entanglement in the melt to the entanglement in the amorphous zone; their experimental results suggest that entanglement controls the thickness of the amorphous zone [36]. Recently, Men et al. revealed the decisive role of entanglements in the transition between the α and tetragonal crystal form of Polybutene-1 [30]. Inspired by these viewpoints, we further investigated the rheology properties of the PLLA samples to analyze the melt state of PLLA before crystallization. Figure 7 shows the rheology data of the five PLLA samples.

Based on the melting temperatures listed in Table 1, the sample temperature of rheology test was 190 °C, and the PLLA could be fully melted. As shown as Figure 7a,b, the storage modulus G′ and loss modulus G″ of these five PLLA samples increases with the increase of frequency ω. With the increase of molecular weight, the G′ and G″ of PLLA increases. This means that molecular weight can obviously influence the rheology properties of PLLA. Furthermore, the high absolute value of the storage modulus means that the molecular chains tend to form the entanglement structure. [37]

In Figure 7c, PLLA2, PLLA3, PLLA4 and PLLA5 show a typical rheology character of thermoplastics polymer: with the increase of ω, shear thinning behavior occurs. The shear thinning behavior of PLLA5 is the most notable, while that of PLLA2 is very weak. These viscosity data mean that, from PLLA2 to PLLA5, the entanglement concentration increases with the increase of molecular weight. The viscosity curve of the PLLA1 sample is almost straight, without shear thinning behavior. The viscosity data means that there is little entanglement in melt PLLA1, and these results basically match with the results of Cooper-White et al. [38].

From the curves of PLLA2, PLLA3, PLLA4, and PLLA5 given in Figure 7d, the elasticity (G″) is significantly larger than the viscosity (G′). With the increase of molecular weight, the slopes of curve decrease. This is because the increase of molecular weight leads to the increase of entanglement, and causes a large energy loss in the process of dynamic measurement. In contrast, for the PLLA1 curve, G′ is almost equal to G″, which shows a Newton flow character.

According to the data published by Dorgan et al., the critical entanglement molecular weight (i.e., Me, molecular weight between entanglements) of PLLA is near 4000 g/mol. (The exact value is 3959 g/mol) [37]. In this section, the rheology properties of five PLLA samples can be explained by Dorgan’s data. The molecular weight of PLLA1 is about 1700 g/mol which is smaller than Me, and there is barely no entanglement in the PLLA1 melt before crystallization. The molecular weight of PLLA2 is about 5500 g/mol, which is slightly bigger than Me, and the PLLA2 melt has a little entanglement. Under external force, the entanglements in the PLLA2 melt can be easily untangled. The molecular weights of PLLA3, PLLA4 and PLLA5 are much bigger than Me, and the entanglement effect is obvious in their melt.

## 4. Discussion

Based on intramolecular nucleation theory [39,40] and molecular nucleation theory [41,42,43,44], a polymer molecular chain tends to aggregate by intramolecular folding in kinetics to reduce the surface of the free energy barrier for nucleation. Miyoshi et al. [45,46,47,48,49] argued that crystallization had a preordered stage of forming cluster nucleus by self-folding. For polymers with small molecular weights, the formation of an integer-fold chain topology structure [50,51] also directly proves that intramolecular self-folding is the preferred crystallization path. For polymers with large molecular weights, the crystallization driving force of arrangement of extended chains or integer-folded chains is needed to extract the molecular chains from the amorphous region. However, the entanglements prevent the timely extraction of the molecular chains, and the molecular chain cannot be arranged into the crystal region by self-folding in a short time. As a result, the molecular chain will form a cluster nucleus by being arranged in parallel with other surrounding chain segments, and then stacked onto the crystal interface [45,46].

The molecular weight of PLLA1 is much smaller than Me, and the PLLA1 melt has no shear thinning phenomenon. So, PLLA1 molecular chains can be assembled into crystals in the form of extended chains or integral folding chains. The molecular weight of PLLA2 is slightly larger than Me, and the slight entanglements are easily untied under the driven force of crystallization kinetics. PLLA2 molecular chains can also be assembled into crystals by integral folding chains. From this point, the formation process of α crystals in the PLLA1 and PLLA2 sample is almost unaffected by entanglement.

By comparison, the entanglement effect is more remarkable in the PLLA3, PLLA4 and PLLA5 samples. With the driving force of crystallization, it is difficult to extract the molecular chain from the entangled network in time. The molecular chain cannot form extended chain and integer-folded chain, but forms non-integer folding chain in the crystal. According to the WAXD results shown in Figure 1, δ crystal is the dominant crystal form in PLLA3, PLLA4 and PLLA5. It is speculated that the formation of δ crystals is closely related to entanglement effect.

As mentioned in Section 3.4, the PLLA3 sample has a character of periodic structure in the SAXS measurement, while the PLLA4 and PLLA5 samples do not, as shown as Figure 6. In the crystalline region, the δ crystal in PLLA3 has same chain conformation with that in PLLA4 and PLLA5. So, the structure or conformation difference of molecular chain in the amorphous region should be considered. During the crystallization process of the polymer chain with entanglement restriction, the intermolecular or intramolecular entanglements are retained in the amorphous region between the lamellae. As a result, tie molecules and loose folds are formed in the amorphous region [23,52]. According to the rheology results shown in Figure 7c,d, the entanglement concentration in the PLLA4 and PLLA5 melts are much larger than that in the PLLA3 melt. In this way, the retained entanglements, tie molecules and loose folds in the amorphous region of the PLLA4 and PLLA5 crystallized samples are more than that of the PLLA3 samples.

Based on the research of Fritzsching et al. [52], entanglements and tie molecules in amorphous regions can cause crowding on the end surface of the lamella. Crowding has multiple effects on the crystal region. Fritzsching et al. [52] proved that crowding on the lamellar surface can reduce the lateral size of the lamella and cause a tilt arrangement of molecular chains in the lamella. In our previous study [23], we also found that loose folds on the crystal surface can lead to lamellar splaying. During the crystallization process, the abundant retained entanglements in PLLA4 and PLLA5 causes serious crowding which can reduce the difference of electron cloud density between the close-packed crystal region and the amorphous region. Hence, it is difficult for the PLLA4 and PLLA5 samples to be measured with long-period data by SAXS. In comparison with the PLLA4 and PLLA5 samples, the crowding effect in the PLLA3 sample may be weaker, and the difference of electron cloud density between the crystal region and the amorphous region is relatively large. As a result, it is possible that the long-period data of the PLLA3 samples can be detected by SAXS.

Furthermore, considering the molecular weight dependence of the transition between δ and α form, the crowded amorphous region may be what hinders the ordering process of the δ molecular helix chain with low mobility under low temperature. When the molecular weight is smaller than the critical entanglement molecular weight, there is little or no crowding on the end surface of the lamella. As a result, the PLLA molecular chains can be assembled in an ordered α system, even though the temperature is below 120 °C. When the molecular weight is significantly larger than the critical entanglement molecular weight, the crowding effect will squeeze the helix chain in the crystal region and the helix chain tends to form disordered δ crystals.

**Figure 8:**
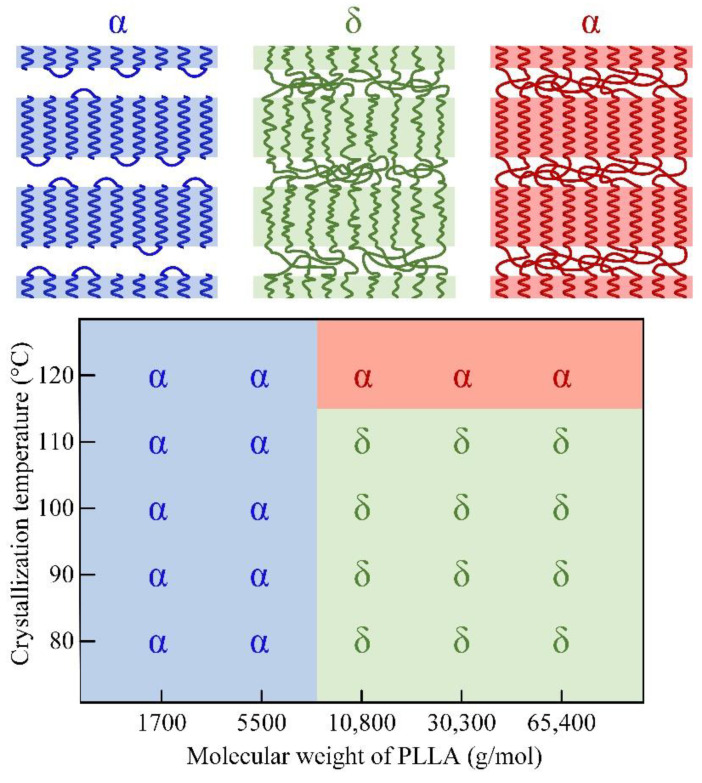
Diagram of crystal forms of PLLA with different molecular weights and different crystallization temperatures.

Based on the above results and discussions, Figure 8 shows a diagram of α/δ crystal forms of PLLA with different molecular weights and different crystallization temperatures. In the view of this research, the low molecular mobility at low temperature and the crowding effect caused by retained entanglements or other amorphous chains are the formation reasons for δ crystals. When the temperature is relatively high, e.g., 120 °C for PLLA with large molecular weights, the crowding effect will also be serious in the amorphous region. However, a molecular chain with high mobility can still form ordered α crystals during the crystallization process. Moreover, when the PLLA sample with δ crystal is heated to a temperature of 120 °C, as shown as Figure 4, δ crystals can be transformed into α crystal without disentanglement, which is a kind of solid–solid phase transition [16,17,18].

Furthermore, during the crystallization of lactic acid-based copolymers, including Poly(l-lactide-co-d-lactide) [53,54], Poly(l-lactic acid-co-l-2-hydroxybutanoic acid) [55,56], Poly(l-lactic acid-alt-glycolic acid) [57] and poly(l-lactic acid-co-l-alanine) [58,59], there is also the phenomenon of crystal form transition. Tsuji et al. studied the polymorphism of poly(l-lactic acid-co-l-alanine) copolymers with wide alanine unit content ranges for melt-crystallization, and the molecular weight of this copolymer is in the range of 10,000~17,000 g/mol. They found that the transition crystallization temperature of copolymer samples from α form to δ form decreases with an increase of alanine unit content [59]. The increase of alanine unit content corresponds to the decrease of the length of the lactic acid unit series, which leads to the decrease of the α/δ crystal transition temperature. In this study, the α/δ crystal transition temperature was decreased by lowering the molecular weight of PLLA, which was consistent with the conclusion of Tsuji et al. [59].

## 5. Conclusions

In this research, five kinds of PLLA with different molecular weights were prepared by ring-opening polymerization with strict dehydration operations and multistep purification treatments. The high purity of l-lactic acid and small polydispersity of PLLA provided us basic conditions to tailor the crystal structure to. The characterization results of WAXD indicated that the PLLA crystal is changed from the δ form to the α form with the decrease of molecular weight at a temperature of 80 °C. Considering the molecular weight effect and rheology feature of PLLA melt, this paper argued that the transition of the crystal form is related to molecular entanglements. The ordering of the δ crystal form may be hindered by crowding on the end surface of the lamella, which is caused by entanglements and other amorphous chain segments at low temperatures. Hence, the transition of the α/δ crystal form is not only associated with temperature, but also related to entanglement state before crystallization. The findings of this research again enhance the significant role of entanglement in polymer crystallization. With significant entanglement effect or molecular weight effect, long-chain molecules of polymer tend to form metastable crystal structures during the ordering process at low temperatures.

## Figures and Tables

**Table 1:** Material properties of five PLLA samples.

Sample	$M_{n}\;(g/\mathrm{mol})$	$M_{w}/M_{n}$	$T_{g}\;({}^{\circ}C)$	$T_{m}\;\;({}^{\circ}C)$	[α]_purified_ (°)
PLLA1	1700	1.18	*	124.9	−151.3
PLLA2	5500	1.22	48.0	152.2	−153.2
PLLA3	10,800	1.14	55.2	166.9	−154.6
PLLA4	30,300	1.23	58.1	173.4	−155.4
PLLA5	65,400	1.26	58.2	174.2	−155.7

* there was no obvious $T_{g}$ step on the DSC curve of PLLA1, given in Figure S3, which was obtained from the secondary heating process from −15 °C to 195 °C.

**Table 2:** The Bragg long periods ($L_{B}$), correlation function long periods ($L_{CF}$), crystal thicknesses ($l_{c}$), amorphous layer thicknesses ($l_{a}$) and transition region thicknesses ($l_{t}$) of the PLLA1, PLLA2 and PLLA3 samples.

Sample Name	$L_{B}\;(\mathrm{nm})$	$L_{CF}\;(\mathrm{nm})$	$l_{c}\;(\mathrm{nm})$	$l_{a}\;(\mathrm{nm})$	$l_{t}\;(\mathrm{nm})$
PLLA1	10.83	11.41	5.15	6.26	1.26
PLLA2	16.11	15.61	6.78	8.83	1.96
PLLA3	14.61	15.25	6.98	8.27	2.11

## Data Availability

The data presented in this study are available on request from the corresponding author.

## Supplementary Materials

The following are available online at https://www.mdpi.com/article/10.3390/polym13193280/s1, Figure S1: 1H NMR characterization results of PLLA1, PLLA2, PLLA3, PLLA4 and PLLA5, Figure S2: GPC curves of the purified PLLA samples, Figure S3: The PLLA sample thickness and surface morphology before crystallization obtained by AFM observation, Figure S4: FTIR profiles of PLLA samples crystallized at 80 °C at wavenumber ranges of 1700–1820 cm^−1^, 1340–1490 cm^−1^ and 1100–1250 cm^−1^, Figure S5: FTIR profiles of PLLA samples crystallized at 80 °C at wavenumber ranges of (a) 1700–1820 cm^−1^ and (b) 1340–1490 ^−1^, (c) 1100–1250 ^−1^.
