# Supplementary material for: Investigation on the α/δ Crystal Transition of Poly(l-lactic Acid) with Different Molecular Weights
Source: Polymers (Basel). 2021 Sep 26;13(19):3280. doi: 10.3390/polym13193280 (PMC8512007; doi:10.3390/polym13193280)
Supplement: Supplementary file 1 [file polymers-13-03280-s001.zip › polymers-1389344-supplementary.pdf]

polymers

MDPI

Supplementary Materials

Investigation on the α/δ Crystal Transition of Poly(L-lactic Acid) with Different Molecular Weights

Lei Zhang, Guoqun Zhao * and Guilong Wang

Key Laboratory for Liquid-Solid Structural Evolution and Processing of Materials (Ministry of Education), Shandong University, Jinan 250061, China; zhanglei_mail@sdu.edu.cn (L.Z.); guilong@sdu.edu.cn (G.W.)
* Correspondence: zhaogq@sdu.edu.cn; Tel.: +86-531-88393238

S1. Measurements methods

Nuclear Magnetic Resonance (NMR). ^{1}H NMR spectrum was recorded on a Bruker AV600 NMR spectrometer using $CDCl_3$ as solvent and tetra-methylsilane (TMS) as internal standard at a proton frequency of 600 MHz.

Differential Scanning Calorimeter (DSC). The glass transition temperature and melting point of PLLA were measured using a NETZSCH STA 4495F5 apparatus. The samples with an average weight of 10 mg is quenched from 190 °C to –20 °C at a rate of 60 °C/min and reheated at a rate of 10 °C/min under a nitrogen atmosphere.

Gel Permeation Chromatography (GPC). GPC characterizations were conducted using Waters GPC system with a 1525 binary HPLC pump, and a Waters 2414 refractive index detector was used. PLLA solutions were prepared in tetrahydrofuran (THF) and prefiltered on filter disk (hydrophobic polytetrafluoroethylene, 0.45 μm pore size) before injection. All measurements were performed using THF as the carrier solvent with a flow rate of 1.0 ml min^{-1} at 30 °C. Standard monodispersed PS (Shodex standard, Kawasaki, Japan) were used for calibration.

Specific Optical Rotation (SOR). The specific rotation value of PLLA was measured at 25 °C in chloroform at a concentration of 0.82 g/dL using a Rudolph spectropolarimeter (Autopol IV-T) with a quartz cell of 100 mm path length at a wavelength of 589 nm.

Fourier Transform Infrared spectroscopy (FTIR). FTIR spectroscopy was carried out using Nicolet 6700/NXR spectrometer (Thermo Fisher) under ATR module. The wavenumber ranged from 4000 cm^{-1} to 400 cm^{-1}, with a resolution of 2 cm^{-1} and a scan number of 64.

S2. Preparation of PLLA with different molecular weights

The pristine PLLA samples (named as PLLA1, PLLA2, PLLA3, PLLA4, PLLA5) were synthesized by ring-opening polymerization, using n-dodecanol and stannous caprylate as initiators and catalysts, respectively. Dispersity is the key parameter to determine the crystal structure. During the synthesis of PLLA, the purity of lactide, the purity of initiator and the water content of solvent are the key indexes that affect the dispersity of PLLA. Water molecules, as well as the active group hydroxyl group will increase the dispersity of PLLA [1]. To minimize water content and hydroxyl content, the following steps need to be taken:

(1) The lactide is purified by recrystallization and finally filtered with dried n-hexane (or petroleum ether),

(2) Stannous caprylate needs to be re-steamed and decompression distillation method is adopted.

(3) distillation treatment should be carried out when the solvent is to be used, the solvent (like methylbenzene) is obtained by distillation process in a dryer,

(4) Before the synthesis, the container was vacuumed through an oil pump, then nitrogen was injected, and then vacuumed for another 30 minutes. The vacuum was repeated three times to completely remove water and oxygen.

In addition, to further reduce the dispersity of PLLA, we performed multi-stage recrystallization treatment on PLLA. PLLA was purified four times by reprecipitation method. In the first three times, the raw PLLA was dissolved with dichloromethane. Then methanol was added to the solution as precipitant and quickly stirred. After adding methanol, continue stirring for 15 minutes. The solution is then filtered through the sand core and the sediment is washed with excessive ethyl acetate. In the last purification step, we use toluene as the solvent. To avoid PLLA degradation caused by hydroxyl group, petroleum ether was selected as precipitant and cleaning agent. Finally, the polymer was dried in a 35 °C vacuum oven for two days.

Figure S1 shows the ^{1}H NMR spectra the five purified PLLA samples. The most intense signals were located at 5.17 and 1.59 ppm which correspond respectively to –CH and $–CH_3$ in the repetitive central units. The peak area ratio between these two groups is about 1 : 3, which matches the number of hydrogen atoms in these two groups. From Figure S1(a), the peak area ratio of the –CH quartet at 5.17 ppm is close to 1 : 3 : 3 : 1 and the peak area ratio of the $–CH_3$ doublet at 1.59 ppm is close to 1 : 1. These characterization results mean that the PLLA samples were prepared.

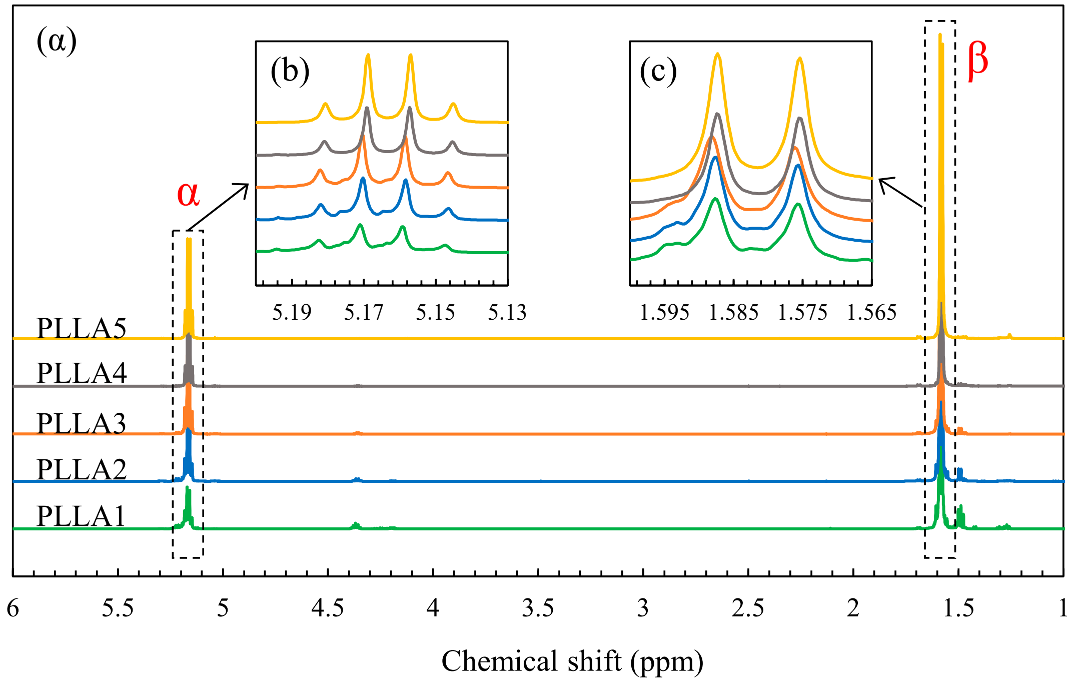

Figure S1. ^{1}H NMR characterization results of five PLLA samples at wavenumber ranges of **(a)** 1–6 ppm, **(b)** 5.13–5.20 ppm and **(c)** 1.565–1.600 ppm. *α* peaks represent the hydrogen proton of –C*H– in the repetitive unit, β peaks represent the hydrogen proton of –C*H_3– in the repetitive unit.

The number-average molecular weight and polydispersity data of the purified PLLA are measured by GPC. The molecular weight data were given in Table 1 and the GPC curves were given in Figure S2. The specific rotation degrees of PLLA samples were listed in Table 1 of main body of this paper. The specific rotation values for the five PLLA samples was found to be in the range of values from –149° to –156°. The L-lactyl contents of PLLA can be evaluated as 100 % as reported in the literature [2,3].

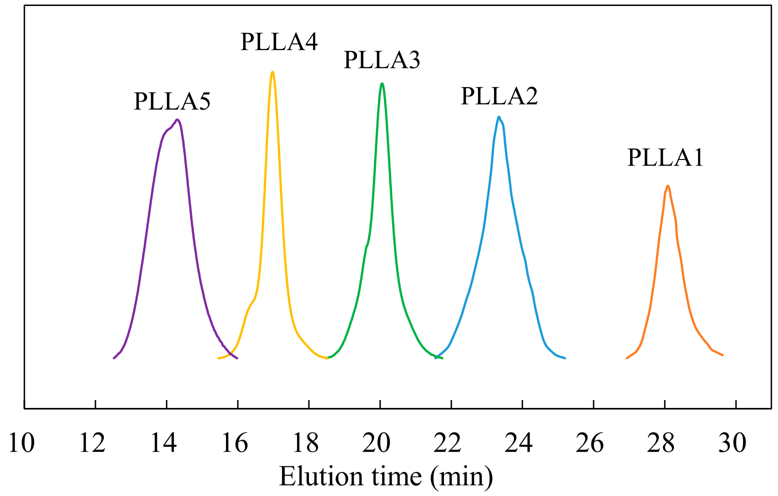

Figure S2. GPC curves of the purified PLLA samples

In addition, the corresponding DSC curves of the purified PLLA samples were given in Figure S3, and the glass transition temperature and melting temperature were listed in Table 1 of main body of this paper. In this study, the thermal properties of PLLA samples were used to set the treatment temperature of crystallization experiments.

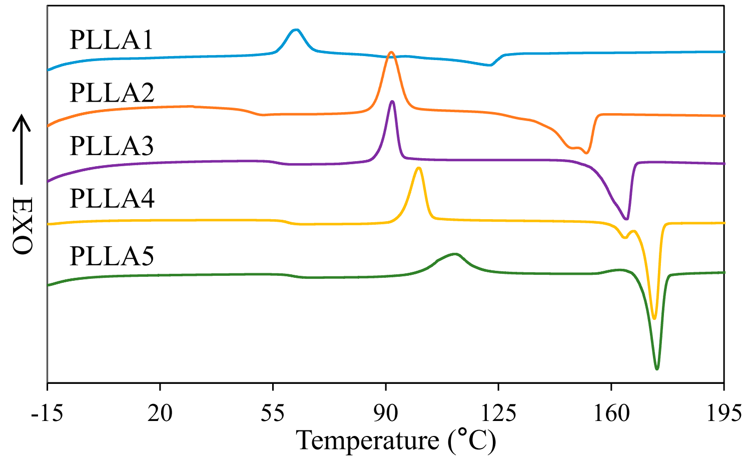

Figure S3. DSC curves of the purified PLLA samples, and the exothermal direction is marked on the Y-axis

S3. Film Preparation

The polymer was dissolved in Dichloromethane at a concentration of 10 mg/ml. Then 20 μl solution was dropped onto a Si wafer which had been cleaned by immersion in Piranha solution in glass ware at 100 °C. To make the Si wafer surface hydrophilic, the Piranha solution was prepared by mixing H_2SO_4 (98%) and H_2O_2 (30%) with a volume ratio of 7:3. A relatively long clean time, 5 hours, was used to enhance the film stability and avoid the dewetting phenomenon at high temperature. Then the boiled substrate was further washed with ultra-pure water. The Si wafer was firstly spun at a rotation speed of 500 rpm for 5 s, and then spun at a rotation speed of 5000 rpm for 40 s by using a KW-4A spin-coater (Institute of Microelectronics, Chinese Academy of Sciences, China). The film thickness is about 180 nm measured by Atomic Force Microscopy, as shown as Figure S4. From Figure S4(c) and (d), the surface of PLLA sample is flat before crystallization, and the maximum fluctuation is only 8.7 nm, which is much smaller than the thickness of the film.

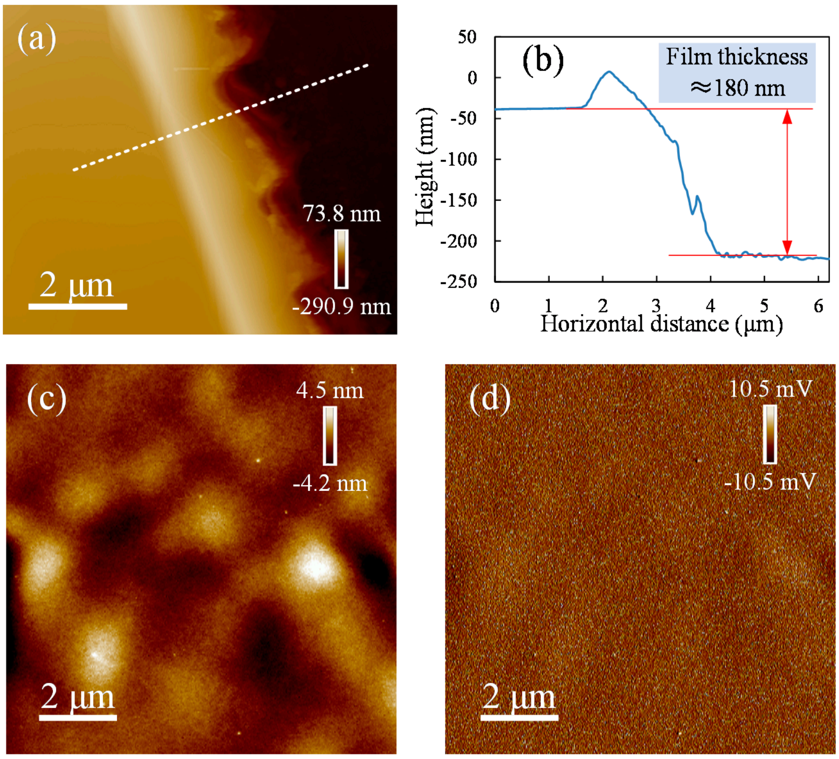

Figure S4. The PLLA sample thickness and surface morphology before crystallization obtained by AFM observation, **(a)** altitude image of PLLA sample near a scratch, **(b)** altitude data on the dotted line in (a), **(c)** altitude image of the surface of non-crystallized PLLA sample, **(d)** peak force error image of the surface of non-crystallized PLLA sample.

S4. Crystallization treatment

The PLLA film coated on a Si wafer was installed in a sample cell of a self-developed in-situ microscopy system [4–6] to implement melt isothermal crystallization. The sample cell was heated to 200 °C. After a holding time of 3 min, the cell was cooled to crystallization temperature by anhydrous alcohol with a temperature of –60 °C. Afterwards, the system temperature was maintained at a constant value to induce crystallization. After isothermal crystallization, the cell was quickly cooled to 20 °C by using the absolute ethyl alcohol with a temperature of –60°C. Finally, the cell was opened and the sample was taken out for subsequent characterization.

S5. FTIR analysis of PLLA samples

In this section, FTIR was conducted to further analyze the effects of molecular weight on the molecular structure of PLLA. Figure S5 show the measured FTIR profiles of the PLLA samples with different molecular weights. The wavenumber with a large difference on the infrared spectrum curves mainly focuses in the range of 1000-1500 cm^{-1} associating with the asymmetric deformation mode of $–CH_3$, C–H bending, C–O–C stretching [7-9] and 1700–1820 cm^{-1} associating with the stretching mode of the C=O group [10-12].

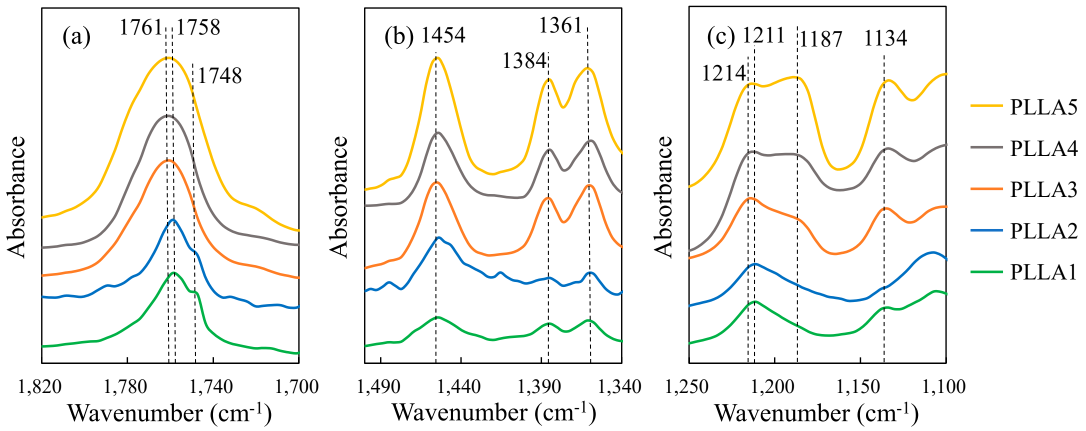

Figure S5 FTIR profiles of PLLA samples crystallized at 80 °C at wavenumber ranges of **(a)** 1700–1820 cm⁻¹ and **(b)** 1340–1490 cm⁻¹, **(c)** 1100–1250 cm⁻¹.

Figure S5(a) depicts the IR spectra of the C=O stretching band (1820–1700 cm^{-1}) for crystallized PLLA samples with different molecular weights. Firstly, the band at 1749 cm^{-1}, which appears only in the spectra of the crystallized PLLA1 and PLLA2 samples, is the characteristic of PLLA α form crystal. In addition, all the five samples show obvious absorption near 1758 cm^{-1} band. The wavenumber corresponding to C=O stretching on PLLA3, PLLA4, and PLLA5 curves are 1761 cm^{-1}, which are relatively bigger than the 1758 cm^{-1} on PLLA1 and PLLA2 curves. This difference means that the energy required to vibrate C=O group in δ crystal is larger than that in α crystal.

In Figure S5(b), the peaks at 1454, 1384 and 1361 cm^{-1} are assigned to the asymmetry bending of $–CH_3$, symmetry bending of $-CH_3$, and symmetry bending of –CH, respectively. These peaks of PLLA3, PLLA4 and PLLA5 samples are stronger than that of PLLA1 and PLLA2 samples. These differences agree with the FTIR results published by Pan [10, 12]. The intensity of IR bands at 1454, 1384 and 1361 cm^{-1} for the δ crystal lower than that of the α crystal.

In Figure S5(c), all the samples show an obvious absorption at 1211–1214 cm^{-1} band which associates with the in-plane rocking vibration of $–CH_3$, indicating the formation of the conformation ordering of backbone [13]. The wavenumber corresponding to $–CH_3$ rocking on PLLA1 and PLLA2 curves is 1211 cm^{-1}, which are relatively smaller than the 1214 cm^{-1} on PLLA3, PLLA4, and PLLA5 curves. This difference means that the energy for backbone stretching vibration of PLLA1 and PLLA2 are smaller than that of other PLLA samples. According to the research of Zhang et al [14], the molecular chain in δ crystal is disordered 10_3 helix, and the δ crystal is relatively looser than α crystal. In this way, the backbones in δ crystal have low stability than that in α crystal to stretch. Furthermore, the peak at 1187 cm^{-1} is assigned to the asymmetric stretching vibration of C–O [13]. In PLLA melting crystallization, the backbone conformation of C–O is sensitive to the helical conformation. Due to the loose assemble of helical chain in δ crystal, longer intermolecular distances usually translate into weak of interactions, resulting in the IR absorption strength of PLLA3, PLLA4 and PLLA5 samples at 1184 cm^{-1} is larger than that of PLLA1 and PLLA2 samples. At this point, the FTIR results also support the judgement about the α and δ crystal of PLLA1-5 samples.

Reference

[1] Liu, Y.; Li, Z.; Sun, L.; Dong, R.; Chen, H.; Xue, Q. Mesophase and Optical Properties of Side Chain Copolyacrylate with Mesogens and OLLA Segments Side Chains, Diffusion and Defect Data. *Solid State Data, Part B. Solid State Phenomena* **2012**, *181-182*, 397–400.

[2] Sarasua, J. R.; Prud'homme, P. E.; Wisniewski, M.; Borgne, A. L.; Spassky, N. Crystallization and melting behavior of polylactides. *Macromolecules* **1998**, *31*, 3895–3905.

[3] Tsuji, H.; Tezuka, Y. Stereocomplex formation between enantiomeric poly (lactic acid) s. 12. Spherulite growth of low-molecular-weight poly (lactic acid) s from the melt. *Biomacromolecules* **2004**, *5*, 1181–1186.

[4] Zhang, L.; Zhao, G.; Wang, G. Investigation on the growth of snowflake-shaped Poly (L-Lactic Acid) crystal by in-situ high-pressure microscope. Polymer 2019, 177, 25–34.

[5] Zhang, L.; Zhao, G.; Wang, G. Investigation on the influence of fold conformation on PLLA lamellar splaying by film crystallization in supercritical CO2. CrystEngComm 2020, 22, 1459–1472.

[6] Zhang, L.; Zhao, G.; Wang, G. Investigation of the Influence of Pressurized CO2 on Crystal Growth of Poly (L-Lactic Acid) by Using In-situ High-pressure Optical System. Soft Matter 2019, 15, 5714–5727.

[7] Lan, Q.; Li, Y. Mesophase-Mediated Crystallization of Poly(L-lactide): Deterministic Pathways to Nanostructured Morphology and Superstructure Control. *Macromolecules* **2016**, *49*, 7387–7399.

[8] Zhang, J.; Duan, Y.; Sato, H.; Tsuji, H.; Noda, I.; Yan S.; Ozaki, Y. Crystal Modifications and Thermal Behavior of Poly(L-lactic acid) Revealed by Infrared Spectroscopy. *Macromolecules* **2005**, *38*, 8012–8021.

[9] Zhang, J.; Sato, H.; Tsuji, H.; Noda, I.; Ozaki, Y. Infrared Spectroscopic Study of CH3•••O=C Interaction during Poly(L-lactide)/Poly(D-lactide) Stereocomplex Formation. *Macromolecules* **2005**, *38*, 1822–1829.

[10] Pan, P.; Kai, W.; Zhu, B.; Dong, T,; Inoue, Y. Polymorphous Crystallization and Multiple Melting Behavior of Poly(L-lactide): Molecular Weight Dependence. *Macromolecules* **2007**, *40*, 6898–6905.

[11] Pan, P.; Zhu, B.; Kai, W.; Dong, T.; Inoue Y. Polymorphic transition in disordered poly (L-lactide) crystals induced by annealing at elevated temperatures. *Macromolecules* **2008**, *41(12)*, 4296–4304.

[12] Pan P.; Han L.; Shan G.; Bao Y. Heating and annealing induced structural reorganization and embrittlement of solution-crystallized poly (L-lactic acid). *Macromolecules* **2014**, *47(22)*, 8126–8130.

[13] Sadtler Research Laboratories. The Infrared Spectra Atlas of Monomers and Polymers. **1980**.

[14] Zhang, J.; Tashiro, K.; Domb, A. J.; Tsuji, H. Confirmation of Disorder α Form of Poly(L-lactic acid) by the X-ray Fiber Pattern and Polarized IR/Raman Spectra Measured for Uniaxially-oriented Samples. *Macromolecular symposia* **2006**, *242*, 274–278.
